# Impacts of the Wild Boar (*Sus scrofa*) on the Livelihood of Rural Communities in Pakistan and Understanding Public Attitudes towards Wild Boars

**DOI:** 10.3390/ani12233381

**Published:** 2022-12-01

**Authors:** Romaan Hayat Khattak, Liwei Teng, Tahir Mehmood, Shakeel Ahmad, Zhensheng Liu

**Affiliations:** 1 College of Wildlife and Protected Areas, Northeast Forestry University, Harbin 150040, China; 2 Institute of Zoology, Guangdong Academy of Sciences, Guangzhou 510260, China; 3 Key Laboratory of Conservation Biology, National Forestry and Grassland Administration, Harbin 150040, China; 4 School of Natural Sciences, National University of Sciences and Technology, Islamabad 44000, Pakistan; 5 Carnivore Conservation Lab, Department of Zoology, Quaid-I-Azam University, Islamabad 45320, Pakistan

**Keywords:** human–wildlife conflicts, *Sus scrofa*, crop raiding, orchard damage, pest animals, questionnaires survey, north-western Pakistan, economic losses

## Abstract

**Simple Summary:**

For minimizing “Human Wildlife Conflicts” (HWCs), it is important to understand the interactions of wildlife with human activities—especially in non-protected areas. The wild boar (*Sus scrofa*) is one of the most widely spread and highly adaptable ungulate species–referred to as a pest species. The increase in wild boar numbers and ranges is linked to the increasing economic and ecological impacts. In Pakistan, wild boar numbers are rapidly multiplying because, generally, it is hunted neither for meat nor for trophies by locals because of strict religious prohibitions. However, in some rural areas, wild boars are killed by the farmers—mostly using firearms—yet, the rate of these kills does not match this animal’s overall reproductive rate. Moreover, a decline in the numbers of apex predators is also adding to the ever-increasing wild boar population. Being a pest species with huge numbers, the wild boar is one of the chief reasons for HWCs in Pakistan. In addition to the economic losses (crop damage and orchard damage) caused by wild boars, a hostile attitude in local communities has developed towards wildlife, in general. In the current study, we investigated the economic effects of the wild boar on pastoral communities’ livelihoods and on locals’ attitudes towards wild boars in northwestern Pakistan. The results revealed that the major crops raided by wild boars were maize, wheat, and vegetables. Most respondents considered the wild boar to be a very common species and wished for its complete elimination from the area. We believe that properly monitoring and controlling the wild boar population, coupled with compensation schemes, can be very promising for minimizing this kind of HWCs.

**Abstract:**

Conservation goals can only be best achieved when there is firm support and cooperation from locals, especially in emerging economies where poor communities often bear most of the cost of human–wildlife conflicts (HWCs). In this study, we explored the economic losses caused by wild boars in two districts, i.e., the Peshawar district and the Nowshera district, in north-western Pakistan. Between May and June 2022, 589 respondents from 53 villages were interviewed. The results revealed that the wild boar was chiefly involved in crop raiding, causing an annual economic loss of USD 12,030 (USD 20.42/household). The highly raided crops included maize (40.24%), followed by wheat (24.95%), vegetables (22.65%), and sugarcane (6.29%). Wild boars were also held accountable for orchard damages. Most people consider the wild boar a common species in the area and want it completely eliminated. We believe that the increasing wild boar population is alarming and should be noticed. The government should collaborate with the local communities to use innovative methods to deter wild boars. Compensation schemes for crop damages should be launched. Moreover, the regular investigation of the wild boar population size and their carrying capacities should be enlisted as integral parts of wildlife management in the area.

## 1. Introduction

Wildlife holds significant value to mankind, comprising ecological, economic, scientific, and spiritual dimensions [1]. However, simultaneously, wildlife may cause damage to human properties and incomes. Any species causing economic losses is usually referred to as a pest animal. This undesirable feature of wildlife is the basic element of the hostile confrontation between humans and wildlife—known as human–wildlife conflicts (HWCs) [2]. HWCs mostly occur in rural areas and are primarily manifested in agricultural areas [3]. A large human population depends on the agricultural areas in developing parts of the world, such as those in African and Asian countries. The populations that exclusively rely on agriculture as their main source of income may lose up to 10–15%, or even more, of their total agricultural output to wildlife [4,5]. Some farmers even face periods of hunger because they produce only enough food to bridge the period until the next harvest [3]. For these communities, wild animals are vermin, resulting in these communities’ generally negative attitudes towards wildlife, thus leading to a decreased cooperation between the agro-pastoral communities and wildlife services.

Pakistan is an agricultural country, accounting for 22.04% of Pakistan’s gross domestic product (GDP) and providing 35.9% of its employment [6,7,8]. In Pakistan, almost 62% of the population is rural—directly or indirectly dependent on agriculture for their livelihoods. Due to Pakistan’s rich biodiversity, several wildlife species are causing damage to agricultural commodities, including crops and livestock [9]. Subsequently, such happenings are typically answered with significant retributory wildlife killings [2,10].

The wild boar (*Sus scrofa*) is a broadly spread ungulate species with remarkable reproductive rates and adaptability [11]. Wild boars are usually involved in causing damage to crops and properties, for which they are referred to as a pest species [12]. Being omnivorous, more than 400 plant species have been identified in its diet, which includes more than 40 agricultural crop plants [13]. As a general image of the agricultural demolition resulting from wild boars, injury events mostly occur in agricultural fields, orchards, forests and pastures, and nurseries [14]. The most alarming aspect of the damage caused by wild boars is the destruction of crops at all stages, from seedlings to mature crops [15]. The exponential increase in wild boars’ numbers and corresponding crop damage caused by them possibly adds much to farmers’ food losses, and this is one of the primary reasons for HWCs [12]. Moreover, in addition to crop damage, wild boars may cause a hostile attitude in the public towards wildlife, in general [2].

In recent years, due to the enormous harmful effects attributed to the wild boar, scientists and wildlife managers have been probing to find effective stoppage and mitigation strategies [16]. Yet, determining the nature of losses and their subsequent economic impacts on the affected rural communities is of the utmost importance. Therefore, the current study was designed to investigate the patterns of crop damages and monetary losses caused by the wild boar in some rural areas of north-western Pakistan and to suggest control measures.

## 2. Materials and Methods

### 2.1. Study Area

**Figure 1:**
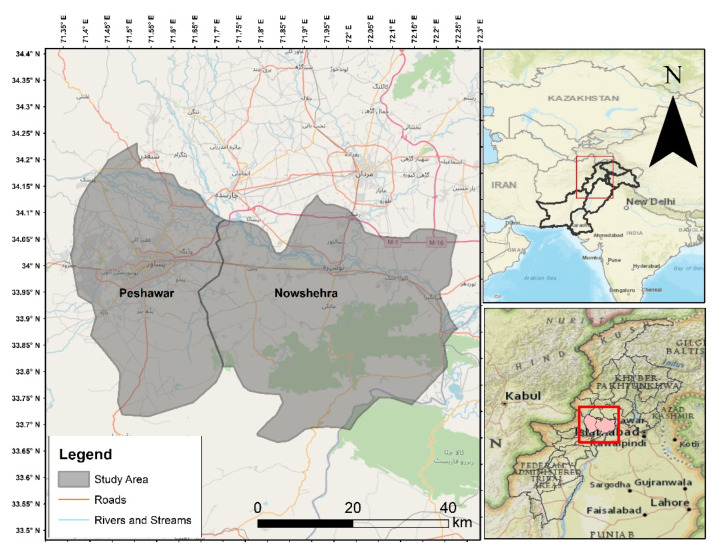
Maps depicting the study areas in northwestern Pakistan.

The current study was conducted within two districts of Khyber Pakhtunkhwa (KP) province, namely, the Nowshera and Peshawar districts (Table 1, Figure 1).

### 2.2. Data Collection

Between May and June 2022, 589 respondents were interviewed from 53 villages in both districts using a semi-structured questionnaire. The questionnaire consisted of two parts. We collected data about the respondent’s basic demographics in the first part. The second part focused on human–wild boar conflicts, including crop and orchard damage and attacks on humans. For crop and orchard damage and attacks on humans, related details such as crop type, type of damage, seasons, victim age, victim sex, and attack location were also recorded. Locals’ attitudes towards wild boars were classified into increase, maintain, decrease, or eliminate; however, the species status was ranked absent, rare, or common. In addition, the number of wild boars killed in the study area during the last two years (2021, 2020) was also noted [9,10].

### 2.3. Analytical Approach

The study area map was developed using Geographic Information System (GIS) ArcMap10.2. Species status, livelihood status, numbers of killed wild boars, and financial damages caused by wild boars were calculated using descriptive statistics in Microsoft Excel 2012.

Principal component analysis (PCA) was used to verify the effects of different factors on crop and orchard damage. Each of these factors was converted by PCA into linear combinations called components [2,9]. For crop damage, we included five factors as inducing patterns of crop damage, i.e., crop type, winter, spring, summer, and autumn losses were considered as follows:

(1) $${\mathrm{PCA}}_{\mathrm{Crop}\;\mathrm{Damage}}=a_{1i}\;\left(\mathrm{Crop}\;\mathrm{Type}\right)+a_{2i}\left(\mathrm{Winter}\right)+a_{3i}\;\left(\mathrm{Spring}\right)+a_{4i}\left(\mathrm{Summer}\right)+a_{5i}\;\left(\mathrm{Autumn}\right)\;$$

For orchard damage, we included five factors as inducing patterns of orchard damage: orchard type, winter, spring, summer, and autumn losses were considered as follows:

(2) $${\mathrm{PCA}}_{\mathrm{Orchard}\;\mathrm{Damage}}=a_{1i}\;\left(\mathrm{Type}\;\mathrm{of}\;\mathrm{Damage}\right)+a_{2i}\left(\mathrm{Winter}\right)+a_{3i}\;\left(\mathrm{Spring}\right)+a_{4i}\left(\mathrm{Summer}\right)+a_{5i}\;\left(\mathrm{Autumn}\right)\;$$

To examine the impacts of wild boars seen, their status, and their numbers and respondents’ household earning members, professions, household sizes, and agricultural land owned on the respondents’ attitudes, we used the logistic regression model (GLM) [2,9,10]:

(3) Attitude=glm(Wild boar Seen+Status+ Wild boar Numbers+ Occupation, Earning Members+ Agricultural Land+ Household Size, family)=‘binomial’

### 2.4. Model Selection

Optimal model (GLM) selection was ensured by using the Akaike information criterion (AIC). To show the influential factors, we used analysis of variance tables. However, the relationship between the significant factors and the response variables was shown by effect plots [10]. The significance level was set at *p* < 0.05, and, analyses were performed in using program R version 4.21.

## 3. Results

### 3.1. Livelihood System in the Study Area

Our results revealed that agriculture was the main source of income for rural communities in the study area. The 589 respondents reported 13,154 livestock owned and averaged 22.33 heads per household. Besides livestock rearing, the locals also grew various crops, vegetables, and fruits. The recorded average farming land owned by each household was 5.90 Kanals.

### 3.2. Sighting Report and Status

Our respondents reported a total sighting of 26,282 wild boars (13,141/year) in the last two years (2020, 2021), with an average yearly sighting of 22.31 per respondent. The majority (99.49%) of respondents declared wild boar a common species in the area.

### 3.3. Human–Wild Boar Conflicts

#### 3.3.1. Crop Damage and Economic Losses

The 589 respondents of the study area thought that the wild boar was accountable for crop damages in the last two years, which translated into a total financial cost of USD 24,060 and a yearly economic cost of USD 12,029 (USD 20.42/household). Most of the crops raided by wild boars were sugarcane, maize, wheat, and vegetables (Table 2). The greatest economic loss caused by wild boars was for maize crops, which was approximately 40.24% of the total crop loss. This was followed by wheat (24.95%), vegetables (22.65%), and sugarcane (6.29%). Orchard damages included uprooted saplings (2.44%), fruits brought down (1.22%), eaten tree trunks (1.08%), and dug-up roots (1.0%).

#### 3.3.2. Human Attitudes toward Wild Boars

Most respondents (96.43%) expressed a hostile attitude towards wild boars and desired their eradication. Approximately 3.39% of the respondents favored reducing wild boar numbers rather than their complete elimination. Only one respondent wished to maintain the current wild boar population in the area.

#### 3.3.3. Consequences of Human–Wild Boar Conflicts

In the past two years, respondents reported killing 702 wild boars (351 per year) in the study area.

### 3.4. Factors Affecting Crop Damage

**Figure 2:**
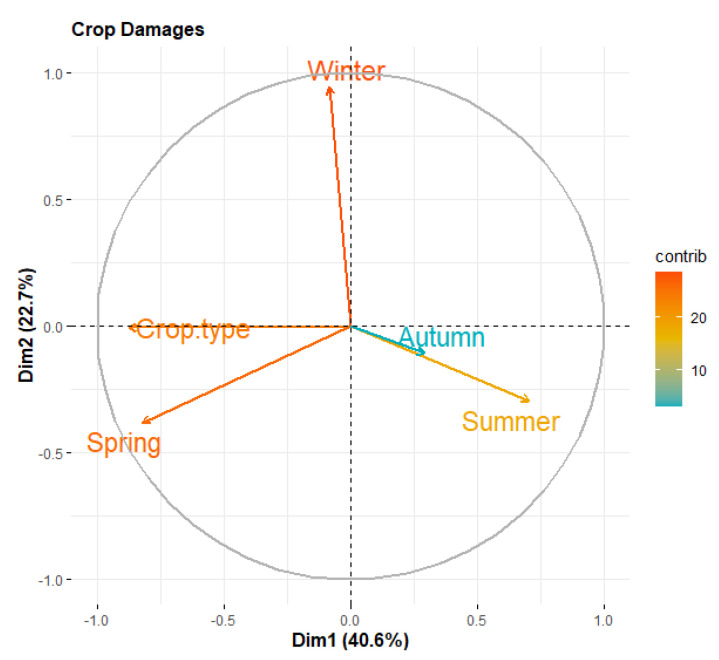
The bi-plot indicating the importance of the crop damage-related factors. The orange color intensity represents the importance of each factor. The first component explains 40.6% of the variations in the total data, while the second component explains 22.7% of the total variations.

The distribution of the five factors considered in the crop damage-based PCA is presented in Table 3 and Table 4. The PCA loadings extracted for crop damage are presented as a bi-plot in Figure 2 for the first two PCA components. The first component, Dim 1, describes 40.6% of the total variations, while the second component, Dim 2, describes 22.7% of the overall variations. The orange color’s intensity shows the significance of each factor in both PCAs. The ‘crop type’, ‘winter’, and ‘spring’ factors were the most significant crop damage features. Summer had a moderate role in crop damage; however, autumn contributed the least (Figure 2). The difference amongst levels is presented in Table 3 and Table 4.

### 3.5. Factors Affecting Orchard Damage

**Figure 3:**
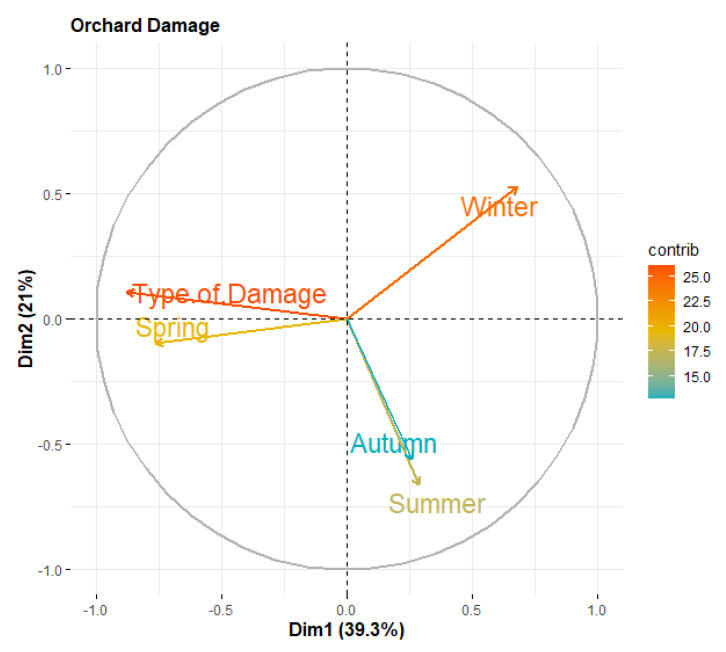
The bi-plot indicating the importance of the orchard damage-related factors extracted by PCA The orange color’s intensity represents the importance of each factor. The first component explains 39.3% of the total data, while second component explains 21% of the total variations.

The distribution of the five factors considered in the orchard damage-based PCA is presented in Table 5 and Table 6. The PCA loadings extracted for the orchard damage are presented as a bi-plot in Figure 3 for the first two PCA components. The first component, Dim 1, explains 39.3% of the total variations, while the second component, Dim 2, explains 21% of the total variations. The importance of the factors over both PCAs is presented by the intensity of the orange color. The ‘type of damage’ and ‘winter’ factors were the most influential factors in orchard damage. ‘Summer’ and ‘spring’ had moderate roles in orchard damage; however, autumn was a minor contributor (Figure 3). The differences amongst the levels are presented in Table 5 and Table 6.

### 3.6. Human Attitudes towards Wild Boars

The effects of some socio-economic factors (shown in Table 7 and Table 8) on locals’ attitudes were investigated by fitting the GLM.

**Figure 4:**
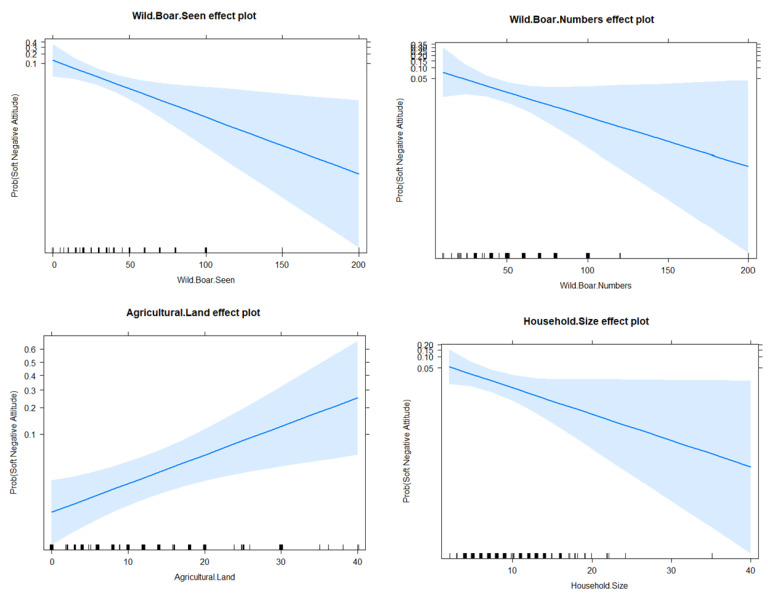
The probability of the appearances of soft negative attitudes for each influential factor, for each level, over the chances of the respondents’ having positive attitudes towards wild boars.

The full GLM model presents the AIC (163.68), while the final fitted GLM model, which kept only the significant factors (with an AIC of 104.41 (Table 9, Figure 4)), revealed that respondents’ attitudes were significantly influenced by four factors: wild boars seen, wild boar numbers, agricultural land, and household size. Two levels of attitude, reduce (soft negative-coded as 1) and eliminate (hard negative-coded as 0), were recorded among the respondents. It appeared likely that with an increase of one wild boar number, the chances of a soft negative attitude were 0.95 times less, with a *p*-value of 0.01. Similarly, with an increase of one wild boar number, the chances of a soft negative attitude were 0.97 times less likely, with a *p*-value of 0.05. With increased agricultural land, the chances of a soft negative attitude were 1.09 times more likely, with a *p*-value of < 0.01. With increase of one household, the chances of a soft negative attitude were 0.84 times less likely, with a *p*-value of < 0.01. These trends are shown in Figure 4.

## 4. Discussion

In the current study, we estimated the financial damages caused by the wild boar in two districts of north-western Pakistan. The results showed that the wild boar is a very common species in the area, with significant numbers, as evidenced by the total annual counts (13,141 per year) reported by the respondents. In Pakistan, the wild boar is hunted neither for meat nor for trophies because of strict religious proscriptions [2]. In addition, the number of apex predators in our study areas is very limited [2,17]. We believe that the reasons mentioned above are contributing to the rapid increase in the wild boar population in the area, and thus compromising the economies of the poor communities.

Wild boars, having large bodies and being highly dependent on plants as a chief component of their opportunistic diet, have great tendencies to trample and consume crops [18]. In addition, when digging and rooting for invertebrate prey and underground plant parts, wild boars cause substantial damage to orchards [18]. The major crops grown in our study area included maize, wheat, different types of vegetables, sugarcane, and fruits. Of all the crops damaged by wild boars, maize had the highest percentage (40.24%), followed by wheat (24.95%), vegetables (22.65%), and sugarcane (6.29%). Overall, these results are in line with those from other studies conducted in Pakistan and across the world, revealing that maize is always a top priority plant for wild boars, followed by wheat [2,14,19,20,21,22]. Wild boars are believed to consume fresh maize as their staple food, even if it is not displaced by mast [18].

The crop damage-based PCA results revealed that the crop damage was highly influenced by the crop varieties and the winter and spring seasons. We assumed that this trend was because of: (1) the most favorable crops (maize) for wild boars, coupled with high-energy yielding crops such as sugarcane, and, (2) being readily available in the study area in the early winter. In addition, in both of the aforementioned seasons, there is a depletion in the natural food resources in the wild [23], thus massively attracting wild boars towards agricultural lands. Likewise, we believe that in spring, a wide variety of vegetables and fruits are available on farms, thus adding greatly to the crop damage perpetrated by wild boars [2]. These results are supported by the findings from Laurent et al. [18], where the relative availability of different food types strikingly determined the overall diet breadth of the wild boar in any instance. Similarly, the results obtained from the orchard damage-based PCA revealed that the type of damage and the winter season were the most influential factors. We believe that this pattern is due to the high consumption of carbohydrate-rich crops (maize and sugarcane) by the wild boar in the winter, which increases the need for animal proteins [24]. Such requirements lead to the uprooting of saplings and digging up roots in orchards in search of invertebrate prey, leading to substantial economic losses [19]. Several aspects of the wild boar’s ecology make them particularly damaging to crops. For example, they raid crops more frequently during the flowering and fruiting seasons. They would continue to raid the same field until they destroyed all its crops [25], thus preventing any chance of replanting the crops and recovering the loss. Further, they do not shy away from forest edges [26,27], providing easy access to farmlands in the human settlements near protected areas. In our study area, most of the agricultural fields were fenceless or surrounded by traditional thorny boma fences, which did not seem to be successful in controlling wild boar infiltration. Electric fencing or net wires are less effective at limiting access for the wild boar than conventional methods, such as scaring devices operated by guards [25,28].

Moreover, given the wild boar’s ability to adapt to deterrents over time, using combinations of different methods that shift over time may be necessary [25]. Therefore, there is a need to move towards more advanced fences and strategies to deter wild boars. Finally, regulated hunting must be considered as the wild boar population grows and their habituation to existing deterrents improves [19,29]. Moreover, the introduction of apex predators (wolves and the common leopard) can be very promising in controlling wild boar populations, as evidenced by an example where wolves managed the unchecked populations of elk (*Cervus canadensis*) in Yellow Stone National Park, USA [30,31,32].

According to the Household Integrated Economic Survey of Pakistan (HIES 2018–19), the average annual income of rural households in KP province is USD 2580 (https://www.pbs.gov.pk/, accessed on 26 October 2021), where crop and orchard losses constitute 9.46% of their annual income. The reported figure of 9.46% in our study is slightly higher than the economic losses caused by wild boars in Nepal, i.e., 9% [33]. A recent study by Khattak et al. [2] revealed that wild boars caused four times more crop damage than porcupines (*Hystrix indica*). Such damages may seem unimportant at state levels; however, given the very low incomes and prevailing poverty in agro-pastoral communities, such losses are substantial to farming households [2]. The economic losses caused by the wild boar are one of the main reasons for the communities’ hostile attitudes towards wildlife, in general.

For designing robust protection and management strategies and enabling improved human–wildlife co-existence, it is very important to understand the attitudes of locals towards the wildlife of an area [34]. The results revealed that in our study area, most locals (96.43%) had negative attitudes towards the wild boar, wishing for its complete elimination from the area. The GLM model revealed that respondents’ attitudes were significantly influenced by four factors: wild boars seen, wild boar numbers, agricultural land owned, and household size (Table 9, Figure 4). It is obvious that the species sightings are directly proportional to their numbers and statuses (common, rare, or absent). Our results showed that the huge numbers of wild boars and wild boars being a common species in the study area were the main reasons for frequent sightings by the locals. It is supposed that the recurrent sightings of wildlife species typically adversely turned the locals’ attitudes, causing a lack of acceptance of wildlife [2], especially if the species were involved in causing significant economic losses. However, the respondents with more agricultural land (≥5 Kanals) showed soft negative attitudes towards the wild boar as compared to the respondents with less agricultural land (<5 kanals) whose attitudes were hard negatives (Figure 4). We attribute this trend to the low agricultural outputs per Kanal relative to the crops and orchard damages caused by the wild boar [2,9,23]. Similarly, the families with larger household sizes (≥7) showed much more negative attitudes towards wild boars as compared to those with smaller household sizes (<7) (Figure 4). These results suggest that the economic losses caused by the wild boars are compromising the economic conditions of low-income families, such that it becomes more difficult for them to meet life’s daily needs, especially in larger households. We believe that the low income levels and the average household sizes (8.6) in our study area (https://www.pbs.gov.pk/, accessed on 26 October 2021) support our findings.

## 5. Conclusions and Recommendations

Our study and earlier studies [2] have documented the wild boar as one of the key drivers of HWCs in north-western Pakistan. Although there is a dearth of wild boar population data and carrying capacity in the study area, the respondents claimed that the population was rapidly increasing and expanding. In response to the economic losses caused by this animal, the retaliatory killing of 702 wild boars took place in the study area. The killing of any wild species in such great numbers indicates the locals’ obvious animosity and hostile attitudes towards wildlife. It is recommended that government and non-government organizations should work together with the local people to exchange ideas and practice innovative methods to deter wild boars. Eliminating some portions of the wild boar population by introducing bounty hunting in the province can be very useful in controlling its rapidly growing population. Farmers should be educated and facilitated regarding more advanced fencing methods for agricultural lands, such as well-maintained big-game-proof fences for successfully reducing wild boars’ infiltration into farming lands and orchards [35]. In addition, investigating the population, carrying capacity, and zoonotic aspects of the wild boar should be an essential part of wildlife management activities. The re-introduction of apex predators (the common leopard and grey wolf) can be very promising for controlling the increasing population of wild boars, thus ultimately reducing the human–wild boar conflicts.

## Figures and Tables

**Table 1:** Study area details with respect to geography, climate, and the human population, along with major crops and key wild mammalian species.

District	Area in km^2^	Population	Urban Population	Rural Population	Annual Average Temperature	Annual Average Rainfall	Major Crops	The Key Wild Mammalian Fauna
Nowshera	1748	1,518,540	339,535 (22.32%)	1,181,460 (77.67%)	24.4 °C	532 mm	Maize, wheat, vegetables, sugarcane, and fruits	Common leopard, grey wolf, red fox, jackal, wild boar, porcupine, and yellow-throated marten
Peshawar	1518	4,331,959	1,969,823 (45.47%)	2,362,136 (54.52%)	22.3 °C	817 mm	Maize, wheat, vegetables, sugarcane, and fruits	Red fox, jackal, wild boar, and porcupine

**Table 2:** Agricultural damages and incurred economic losses due to wild boars in the study area during the years 2020 and 2021.

Crop Types	Total Loss USD	Loss in PKR
Sugarcane	1514	245,296
Wheat	6002	972,318
Maize	9682	1,568,485
Vegetables	5449	882,733
Sorghum	31	5061
Bringing fruits down	295	47,713
Digging roots	240	38,931
Eating tree trunks	261	42,214
Uprooting saplings	586	94,947
Total loss	24,060	3,897,699
Annual loss	12,029.94	1,948,849.50
Per hh/year loss	20.42	3308.74

hh = household; 1 USD = 162 PKR.

**Table 3:** Crop damage (*n* = 694) by wild boars used in the crop damage-based PCA. The values indicate the number of crop damages for each level (counts) and the percentages (%) for each categorical factor used in the crop damage-based PCA.

Crop Damage	Counts	%
Maize	308	44.38
Melons	14	2.02
Sorghum	2	0.29
Sugarcane	56	8.07
Vegetables	125	18.01
Wheat	189	27.23

**Table 4:** Continuous factors associated with the crop damage (*n* = 694) by wild boars used in the crop damage-based PCA. The values shown include the means, medians, standard deviations, minimums, and maximums of the factors used in the crop damage-based PCA.

	Minimum	Mean	Median	SD	Maximum
Winter	3000	42,851.35	40,000	22,333.51	1.00 × 10^5^
Spring	2000	58,639.18	50,000	32,917.82	4.00 × 10^5^
Summer	2000	42,188.41	40,000	30,925.21	450,000
Autumn	199	38,242.1	20,000	93,260.11	1,400,000

**Table 5:** Orchard damage type (*n* = 130) by wild boars used in the orchard damage-based PCA. The values shown include the number of crop damages for each level (counts) and the percentages (%) for each categorical factor used in the orchard damage-based PCA.

Type of Damage	Counts	%
Bringing Down Fruits	7	5.38
Digging Roots	17	13.08
Eating Tree Trunks	12	9.23
Uprooting Saplings	94	72.31

**Table 6:** Factors associated with the orchard damage (*n* = 130) by wild boars used in the orchard damage-based PCA. The values shown include the means, medians, standard deviations, minimums, and maximums of the continuous factors used in the orchard damage-based PCA.

	Minimum	Mean	Median	SD	Maximum
Winter	1000	41,593.75	30,000	47,209.12	200,000
Spring	1000	11,266.3	10,000	7701.666	40,000
Summer	3000	50,600	10,000	84,123.72	200,000
Autumn	30,000	30,000	30,000	NA	30,000

**Table 7:** Factors associated with attitudes towards wild boars (*n* = 589). The values shown include the means, medians, standard deviations, minimums, and maximums of the continuous factors used in the GLM model.

	Minimum	Mean	Median	SD	Maximum
Wild boars seen	0	44.5365	40	24.31932	200
Wild boar numbers	10	55.36503	50	19.56354	200
Age	18	41.41935	42	9.711083	70
Earning members	1	2.062818	2	1.053562	12
Agricultural land	0	5.908319	0	7.987985	40
Household size	2	8.789474	9	3.461129	35

**Table 8:** Categorical factors used in the GLM model associated with the attitudes towards wild boars (*n* = 589). The values shown include the number of respondents for each level (counts) and the percentages (%) for each factor.

Factor	Value	Count	%
Status	Common	583	98.98132
	Rare	6	1.018676
Attitude	Eliminate	568	96.43463
	Maintain	1	0.169779
	Reduce	20	3.395586
Education	Graduate	167	28.35314
	Illiterate	77	13.07301
	Primary	345	58.57385
Occupation	Business	88	14.94058
	Employee	203	34.4652
	Farmer	197	33.44652
	Labor	75	12.73345
	Student	26	4.414261

**Table 9:** The effects of the significant socio-economic factors on the attitudes of locals towards wild boars. The estimate is the GLM-based effects translated by the logs as odds ratio (standard error). The significance of each level compared to another level (called reference level) is presented by the *p*-values, which are further supported by the *z*-values.

	Odds Ratio	Estimate	Std. Error	*z*-Value	*p*-Value
Intercept	2.67	0.98	1.22	0.81	0.42
Wild boars seen	0.95	−0.05	0.02	−2.54	0.01
Wild boar numbers	0.97	−0.03	0.02	−1.71	0.05
Agricultural land	1.09	0.09	0.03	2.92	<0.01
Household size	0.84	−0.18	0.09	−1.98	0.05

## Data Availability

All of the data obtained are presented in this article.
